# A Successful Non-microsurgical Thumb Reconstruction by Using the Skeletonized Amputated Phalanges, After a Failed Trial of Microsurgical Replantation: A Case Report

**DOI:** 10.7759/cureus.32377

**Published:** 2022-12-10

**Authors:** Maha Hanawi, Tanveer A Bhat, Hesham Alokaili, Abdulla Altamimi, Mohammed Y Mirza

**Affiliations:** 1 Department of Plastic and Reconstructive Surgery, King Saud Medical City, Riyadh, SAU

**Keywords:** skeletonized, microvascular, hand reconstruction, replantation, digital amputation

## Abstract

We present a case of thumb reconstruction free of microsurgical technique or free tissue transfer producing satisfactory function. The patient who underwent reconstruction is a 40-year-old right-handed male mechanic, medically free, non-smoker. After unsuccessful microsurgical replantation of the amputated thumb, we elected to pursue regional reconstruction options using a reverse radial forearm flap and the skeletonized phalanges obtained from the amputated thumb as a bony scaffold.

## Introduction

A complex mechanism among joints, muscles, tendons, ligaments, and fascia gives to the thumb its paramount function and worthy replantation and reconstruction methods postamputation. In previous studies, the thumb function quantified to be almost 40% of the total human hand function [1].

The thumb consists of only two phalanges, yet it has a greater range of motion than all other fingers; flexion, extension, abduction, adduction, opposition, and retropulsion. The level of thumb amputation is generally classified as distal to the interphalangeal (IP) joint and proximal to the interphalangeal (IP) joint. The latter region can be further subdivided into subtotal amputation which occurs proximal to IP joint and total amputation which takes its place proximal to proximal phalanx [2].

Preserving the thumb's length, function, sensation, and appearance are all essential goals in thumb replantation versus reconstruction. Some factors affecting the intervention plan are related to the patient such as patient preference, hand dominance, age, sex, occupation, and smoking history. Other factors are related to the injury itself, for example, mechanism of injury, level of amputation, and time. In addition, availability of microsurgical instruments and surgeon experience play an important role in decision-making [2-4].

The choice of thumb replantation using microsurgical techniques is always a priority over reconstruction, if applicable, otherwise reconstruction should be taken into consideration [1,2]. According to previous studies, successful replantation rates of total and subtotal amputations vary among institutions from 55% to 93% [4]. Arterial and venous thrombosis have been considered the most common complications post-replantation [4].

Inversely, when replantation is not feasible, multiple reconstructive techniques for proximal thumb amputation, i.e., proximal to IP joint have been established and succeeded, for example, toe-to-thumb transfer, web space deepening using flaps or skin grafts, metacarpal lengthening, pollicization, and osteoplastic reconstruction using bone grafting and flaps [1-3].

Osteoplastic reconstruction is a procedure that has been used for subtotal thumb amputations with intact carpometacarpal (CMC) joints in two steps; harvesting of cortico-cancellous bone grafts from the iliac crest which are then inserted in a tubularized pedicled groin flap followed by dividing the flap for tissue coverage and debulking procedures. That procedure may result in a good thumb length and soft-tissue coverage, however, it has multiple drawbacks one of which is donor site morbidity [1,3].

To the best of our knowledge, a limited number of studies have been done regarding thumb reconstruction using the amputated part as a bone graft rather than harvesting from the iliac crest or radial bones. Hereby, we present a case of a mangled hand post-machine injury in a 44-year-old right-handed male patient who underwent a successful thumb reconstruction by using the skeletonized phalanges of the amputated thumb as bone grafts for skeletal support, after a failed microvascular replantation.

## Case presentation

**Figure 1:**
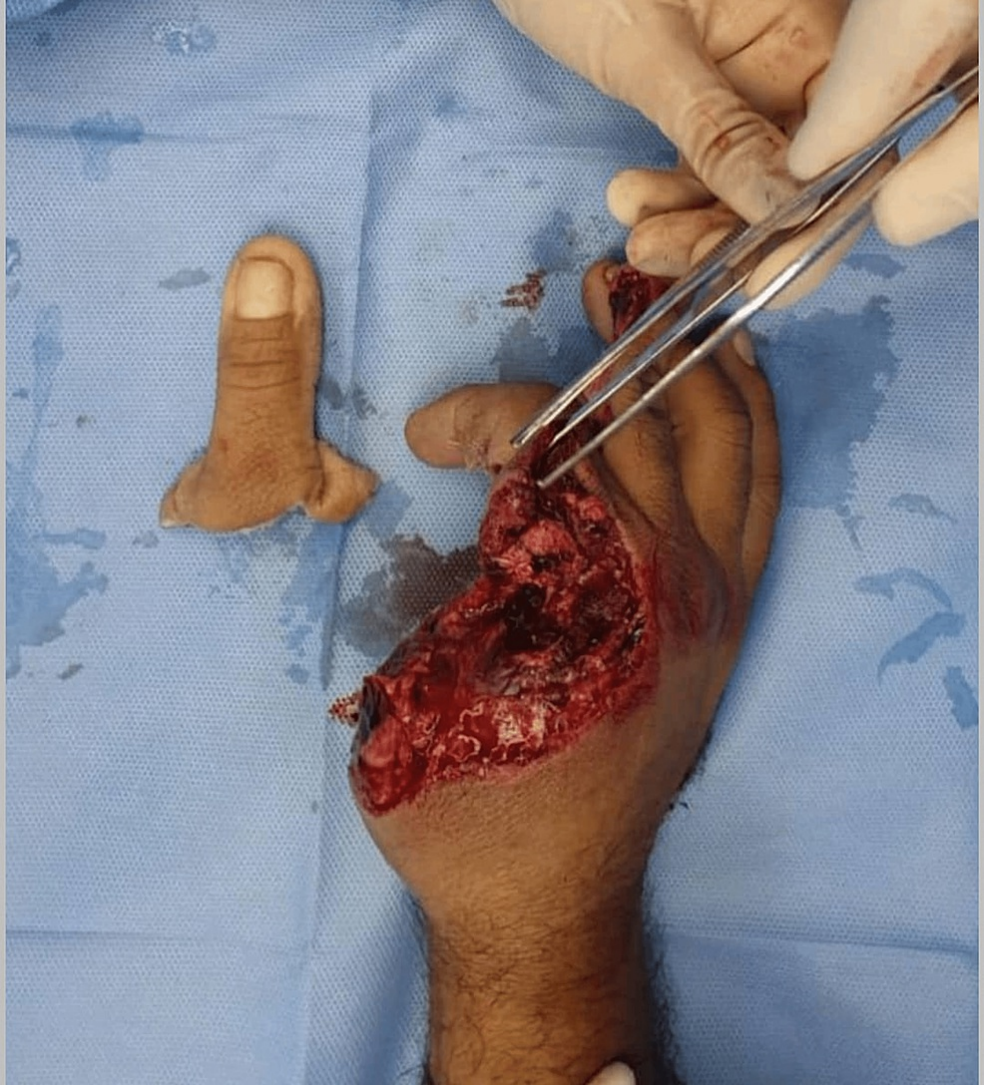
Total amputation of the thumb

A 40-year-old right-handed male, medically free, nonsmoker, and worker attended our emergency department as a case of right-hand machinery injury with total amputation of the thumb and trauma to volar aspects of the index and middle fingers (Figure 1). The patient sustained an injury while operating a woodworking machine and afterwards reported to the hospital 5 hours post trauma. While giving the first aid, the patient was simultaneously evaluated for any life-threatening injuries according to the latest advanced trauma life support (ATLS) protocol by the emergency physicians. The patient received a single intramuscular dose of tetanus toxoid, broad-spectrum intravenous antibiotics, and multimodal analgesia. Hand X-rays were ordered and blood samples were sent which comprised a complete blood count (CBC), coagulation profile, kidney function tests, liver function tests, and blood grouping with cross-matching. Local examination revealed complete amputation of the thumb at the metacarpophalangeal (MCP) level with surrounding soft tissue loss involving the stump of the thumb, extending to the dorsum of the hand, and also involving the volar aspects of the proximal third of the index and middle fingers. The hand X-ray showed amputation of the thumb through the MCP joint with loss of the radial half of the second metacarpal head.

After properly documented consent, the patient was shifted to the emergency operation room. He underwent exploration of the wound and reassessment of the amputated thumb for possible replantation. Initially, the patient was given an axillary block which was later converted to general anesthesia with orotracheal intubation. Debridement and exploration of the wound was done under tourniquet control followed by the assessment of the amputated thumb for replantation.

**Figure 2:**
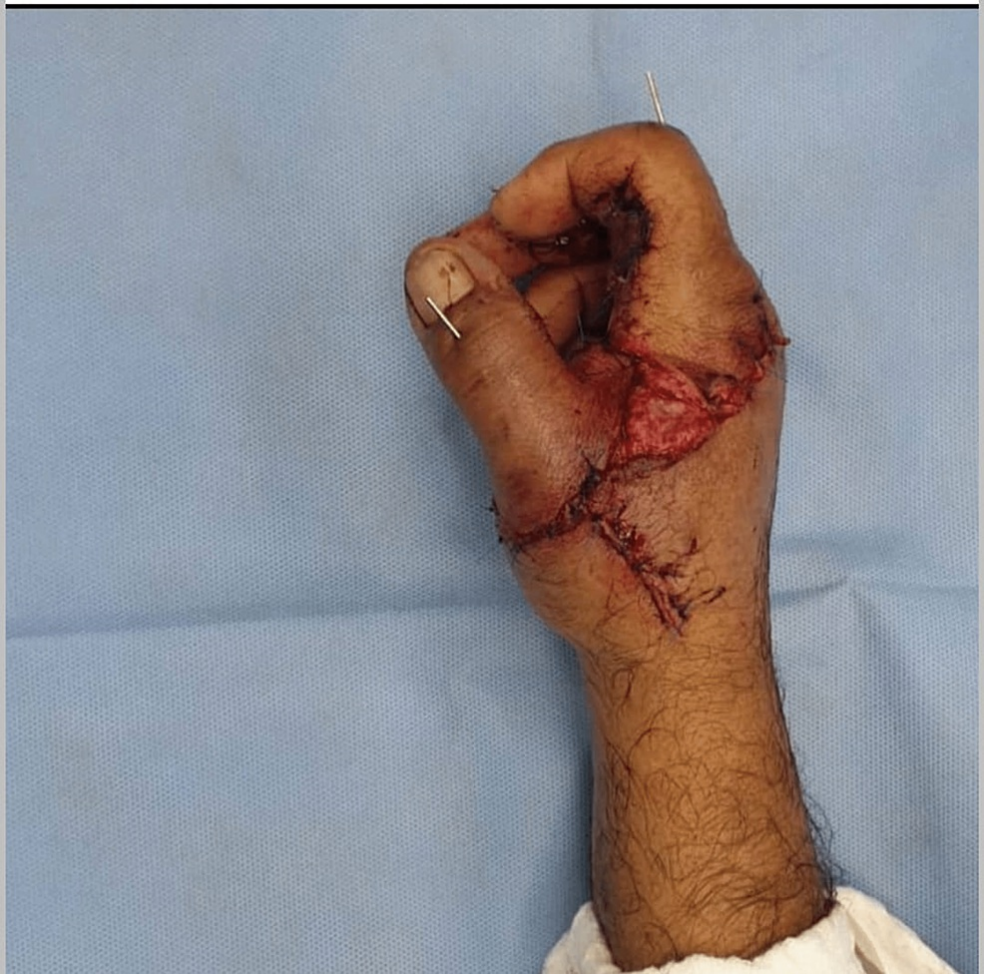
Replanted and fixated thumb

**Figure 3:**
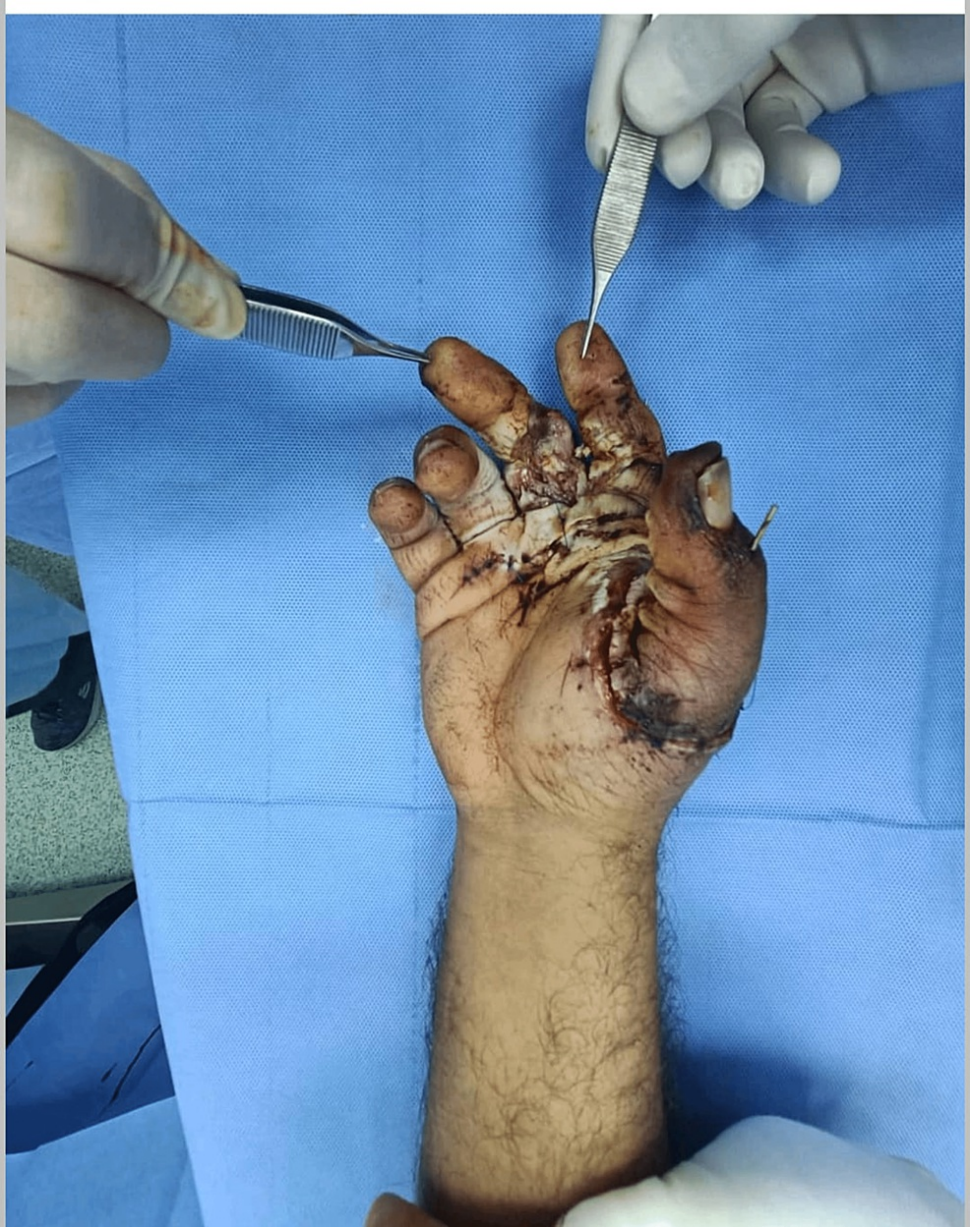
A non-viable thumb post replantation failure

Replantation of the thumb was completed successfully by doing microvascular end-to-end anastomosis of two arteries and three dorsal veins using 9-0 prolene under an operative microscope (Figure 2). On the second post-operative day, the patient developed arterial insufficiency for which immediate re-exploration was performed. Both the anastomotic arteries were found thrombosed intra-operatively distal to the anastomotic site. Revision of arterial repairs was done. However, the vascularity of the thumb did not improve due to recurrent thrombosis as a complication of crush injury. Thereby resulting in the failure of the procedure (Figure 3).

**Figure 4:**
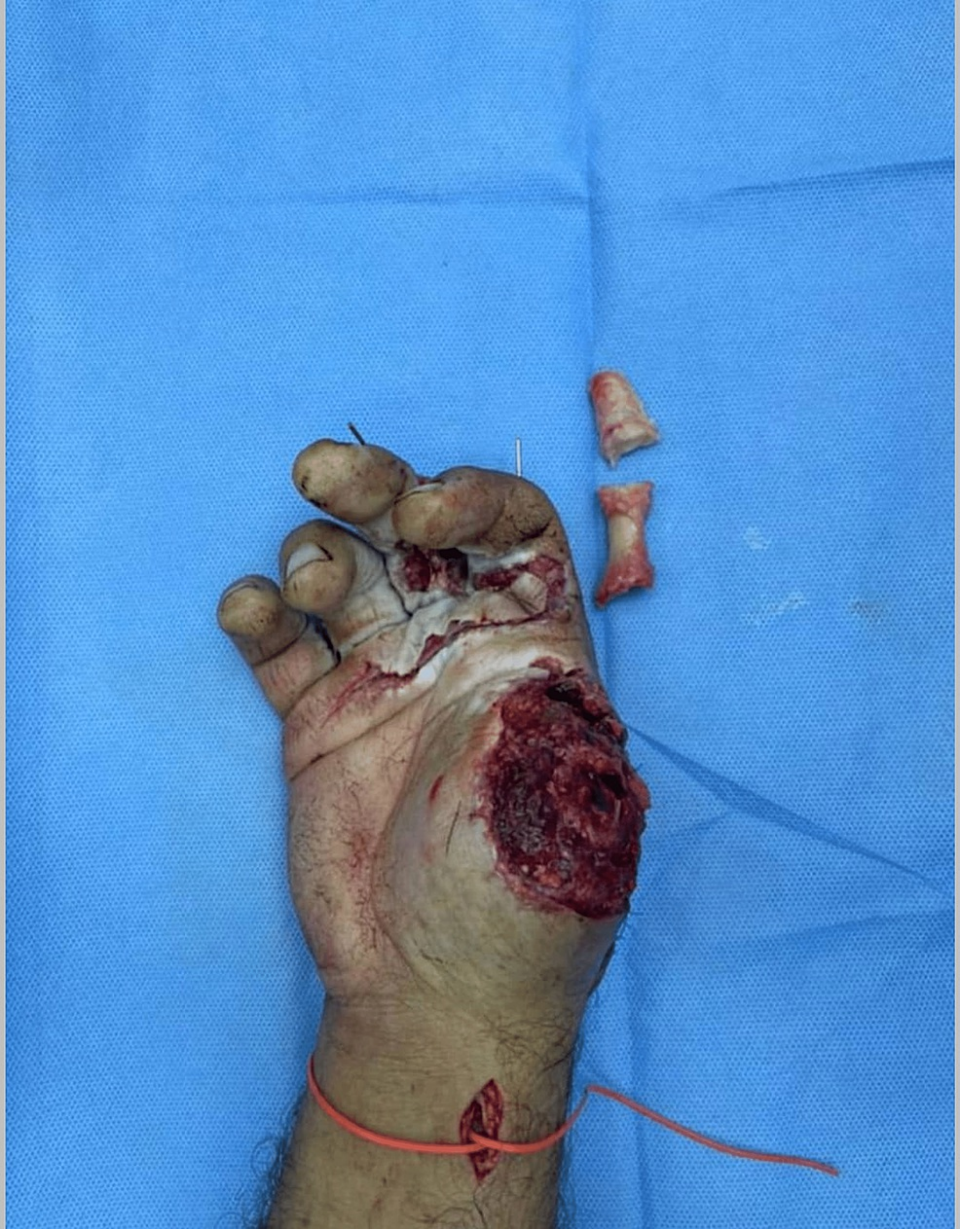
Skeletonized phalanges of the amputated thumb

**Figure 5:**
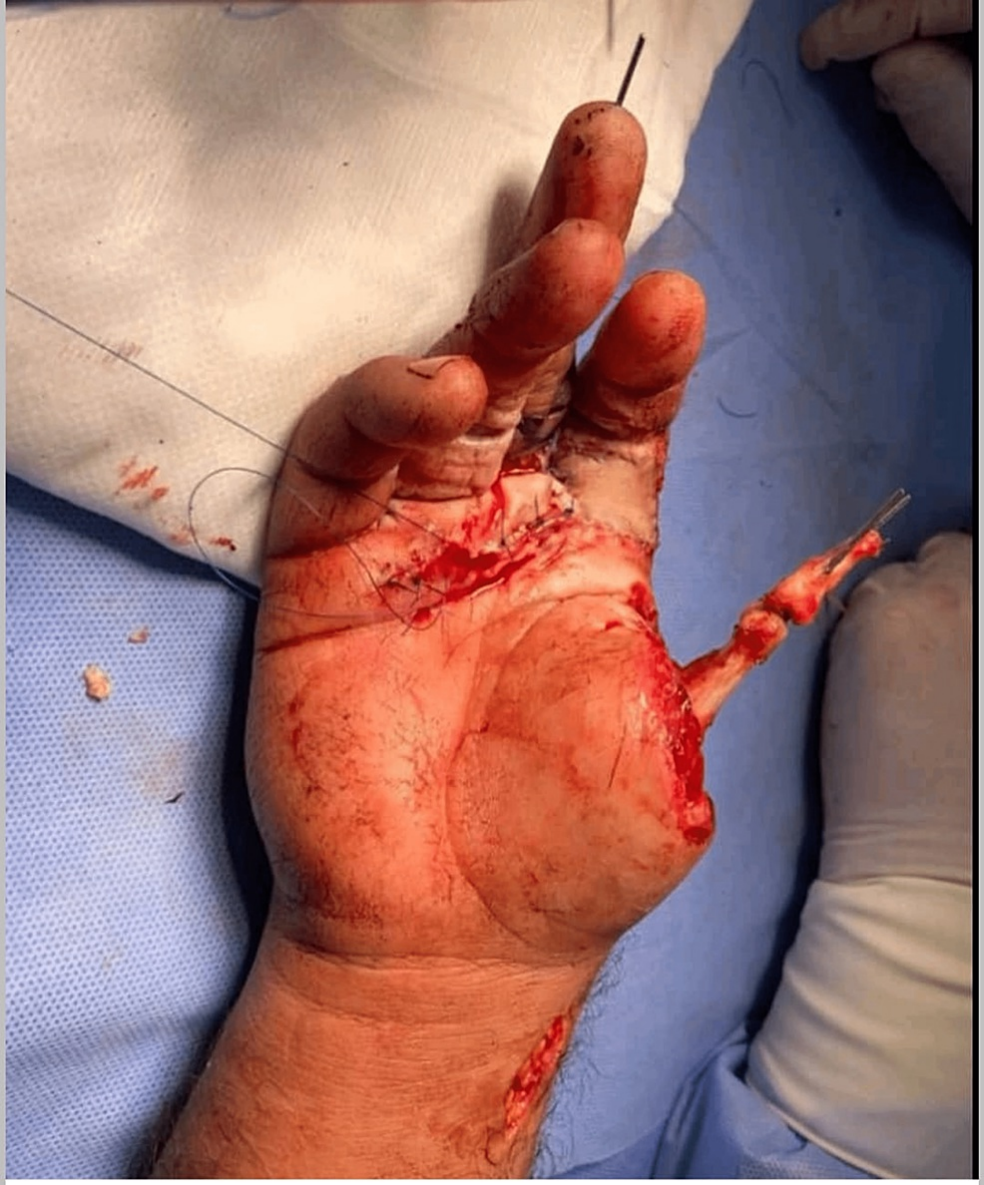
Fixation of the skeletonized phalanges with the thumb metacarpal using two axial k-wires

**Figure 6:**
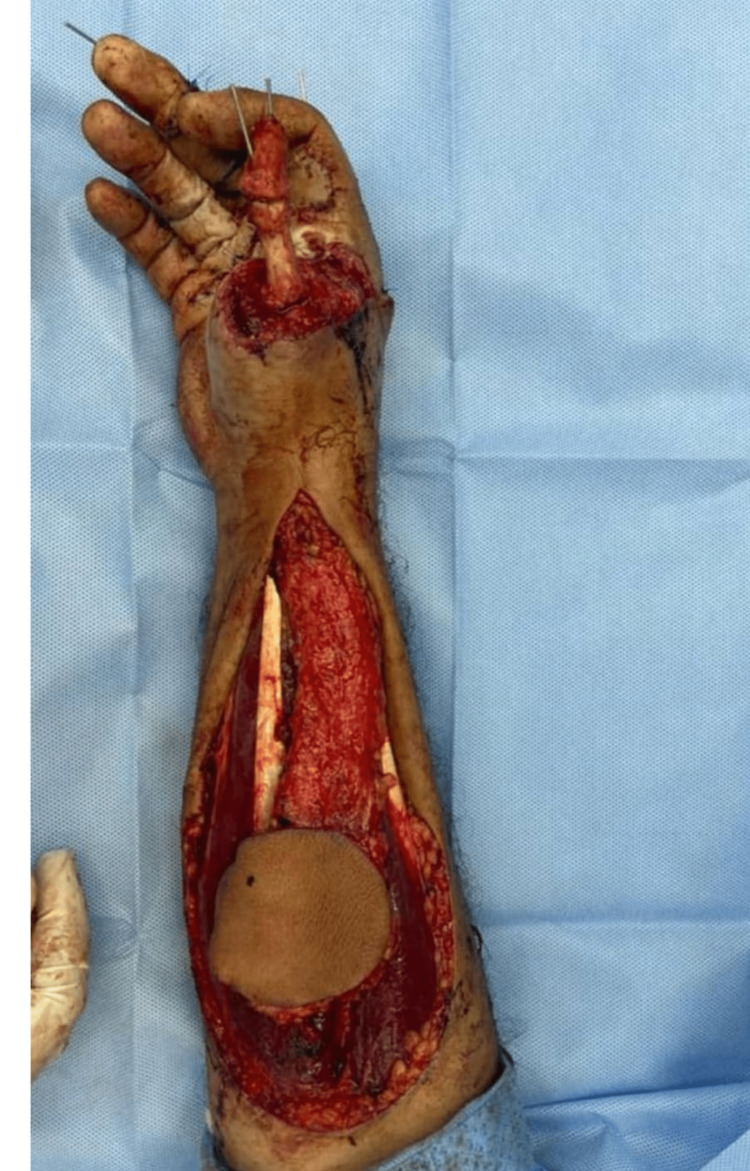
An islanded reverse radial forearm flap

**Figure 7:**
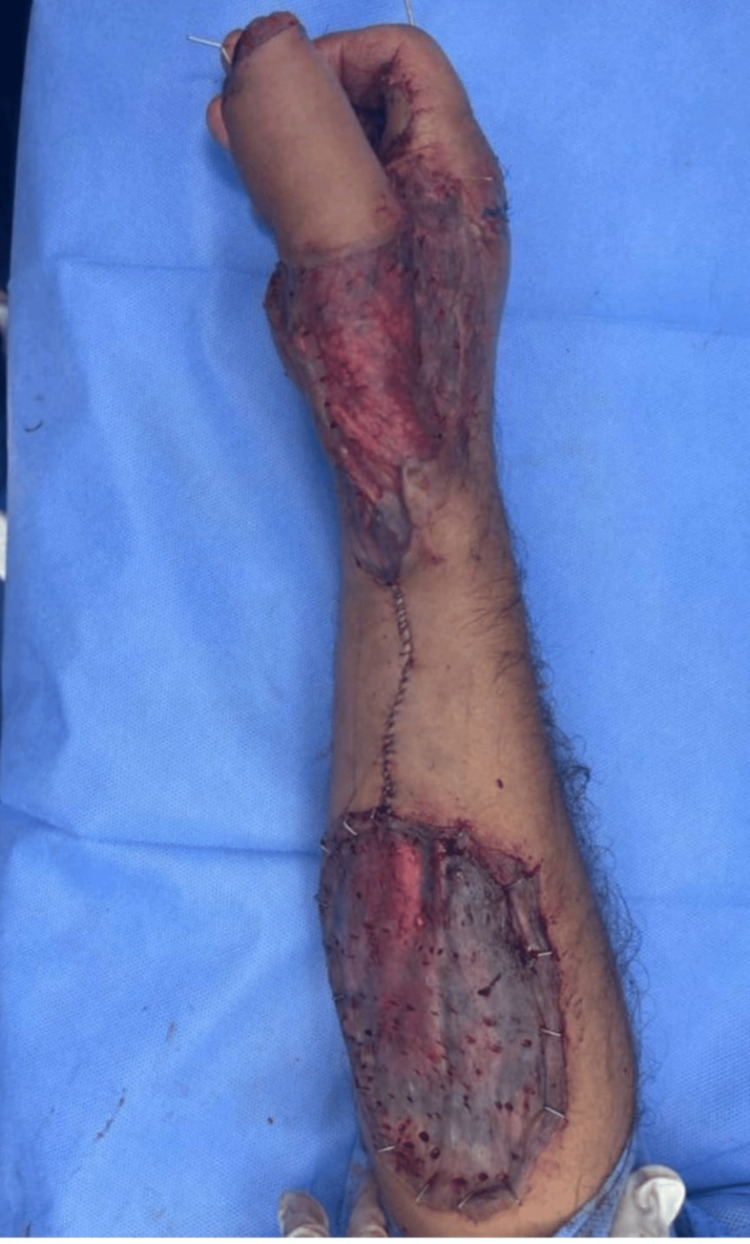
The reconstructed thumb with coverage of the skeletonized phalanges by the reverse radial forearm flap

On the fifth post-operative day, a proper consent was taken after discussion with the patient about the further management plan regarding the immediate reconstruction of the thumb. Preoperatively, Allen’s test was done using a hand held Doppler to confirm the patency of the palmar arch for harvesting the reverse radial forearm flap (RRFAF). Under general anesthesia and tourniquet control, thorough debridement of the non-viable tissue was done, followed by skeletonization of the amputated thumb phalanges. Afterwards, excision of the articular cartilage was performed and leaving the periosteum attached to the bones in order to facilitate graft take (Figure 4). Two axial k-wires sized 1mm were used to fix the bone grafts to the metacarpal of the thumb (Figure 5). A 12×8 cm² reverse radial fasciocutaneous flap was designed on the forearm to cover the bone grafts (Figure 6). After that, the flap was raised in the subfascial plane as an islanded flap. The donor site and the pedicle of the flap were covered with a split-thickness skin graft (STSG) harvested from the left thigh (Figure 7). Subsequently, a cross-finger flap raised from the ring finger was used to cover the exposed flexor tendons of the middle finger. The donor site and the soft tissue loss over the index finger were covered by a full-thickness skin graft (FTSG) harvested from the groin. At the end of the operation, the non-adherent dressing was used over all operative sites. Finally, a thumb spica was used to splint the thumb. The first dressing change was done on the fifth postoperative day and the graft was well-taken. The cross-finger flap detachment with final insetting was done after three weeks.

**Figure 8:**
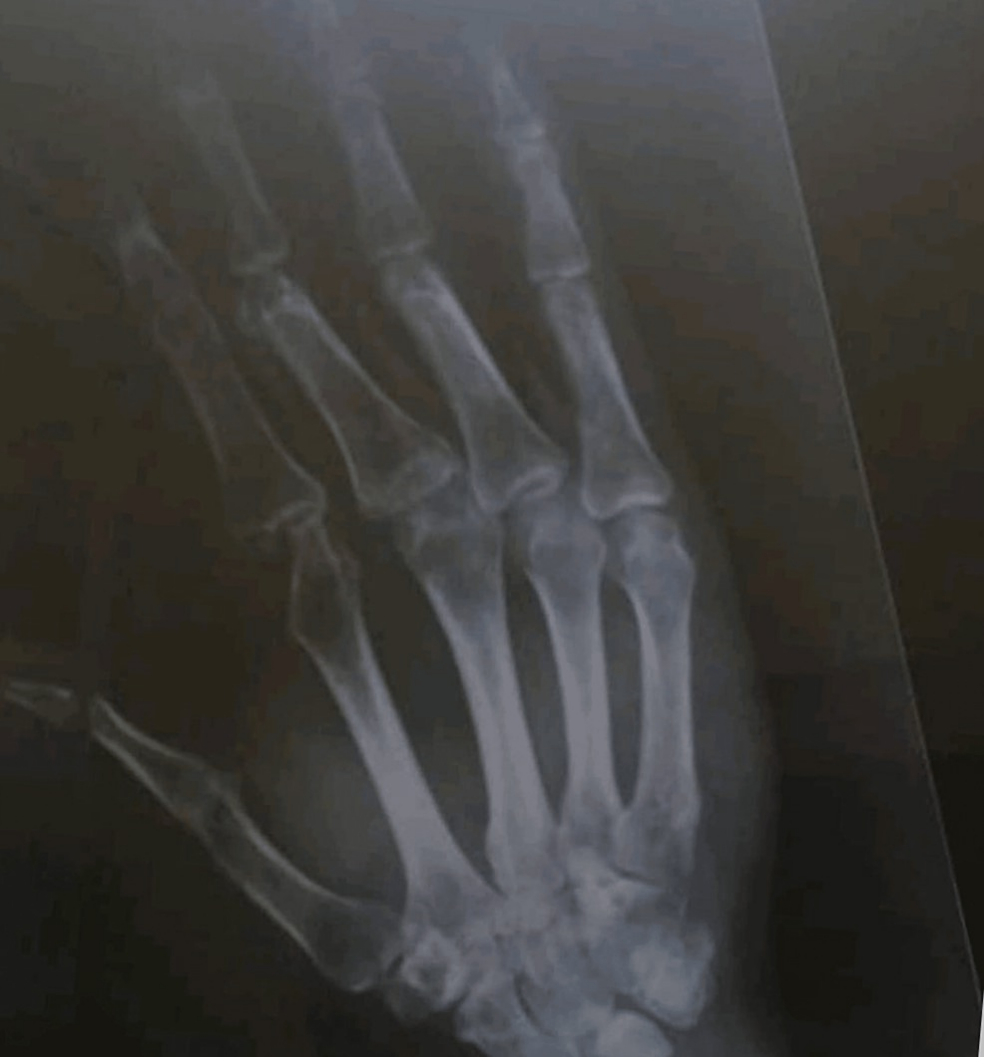
Non-digital radiograph of the hand showing preserved bone grafts at 11 months post-reconstruction

**Figure 9:**
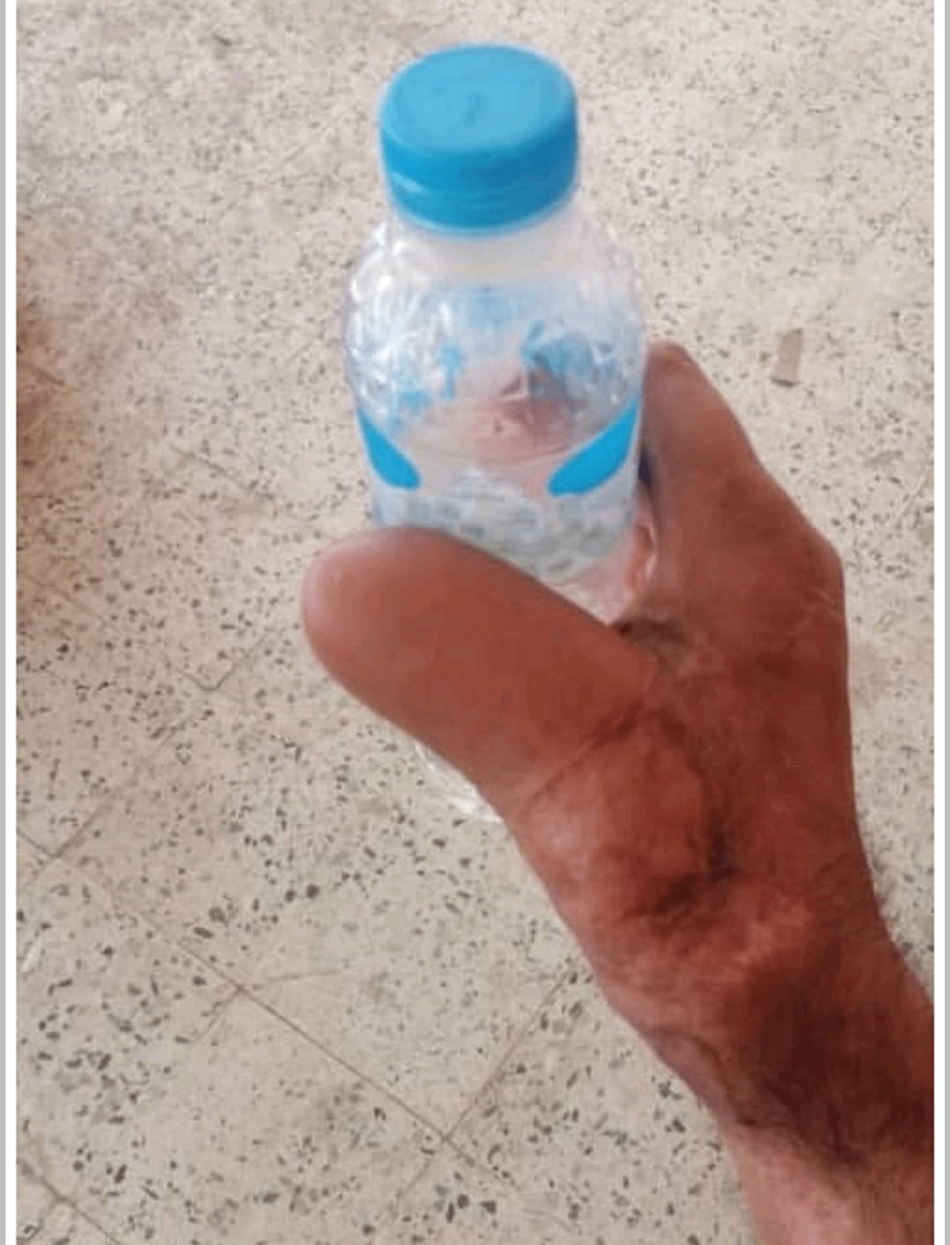
The stable reconstructed thumb with a good grasp

At follow-up, X-ray of the hand was repeated at two months and later at one year which showed intact bone grafts without any signs of bone resorption (Figure 8). Though the patient’s reconstructed thumb has no MCP joint, the presence of the intact functional CMC joint with its attached tendons gave the patient some range of motion, and acceptable opposition and grasping (Figure 9), which satisfied the patient. Later on, the patient was offered secondary procedures in the form of soft tissue debulking and reconstruction of the pulp with a neurosensory flap, but he refused as he had already returned to work. Fortunately, his occupational demands are well-served with his hand and he is content.

## Discussion

As the thumb function is previously estimated to be 40% of the hand function, thumb replantation or reconstruction is a necessity. Our patient presented to the emergency department as a case of mangled right hand with amputation of the thumb at metacarpophalangeal level 5 hours post trauma, which is considered within the acceptable time window for digit replantation [4]. A trial of thumb replantation was performed which failed because of the arterial inflow insufficiency secondary to the recurrent arterial thrombosis. Complications that occur following thumb replantation are numerous, the commonest of which is indeed arterial and venous thrombosis. Though smoking is considered a potent risk factor for the previous complication, our patient developed arterial insufficiency regardless of being a nonsmoker. Our decision to take the patient to the operating room for reconstruction was not only based on the surgeon's preference, but also on our patient’s age, sex, occupation demands, time of injury, and level of amputation which were all favorable factors.

Thumb reconstruction is a method that may be considered if replantation is not possible. The technique determined is based on the level of amputation which can either be proximal or distal to the interphalangeal joint. Many techniques have been used previously and succeeded for proximal thumb amputation. For instance, web space deepening using flaps or skin grafts, metacarpal lengthening, pollicization, and osteoplastic reconstruction, toe-to-thumb transfer, the latter has been the gold standard technique. The toe-to-thumb transfer may result in an excellent cosmetic outcome and precise function, but its long-term recovery and donor-site morbidity are inevitable [1]. Pollicization, in addition, is another good option in patients with total amputation of the thumb with a functional IP joint, however, it does not result in a good aesthetic look [1].

On the contrary, it was vivid from the literature that osteoplastic reconstruction has multiple advantages over the previously mentioned strategies. It is a non-microsurgical operation where it can be done without the need of micro-surgical experts. First, an autologous bone graft is usually harvested from the iliac bone or radial bone followed by soft tissue coverage with a groin or forearm flap and ultimately a neurovascular island flap should be taken to provide sensation to the thumb [1]. Although these procedures may result in a good thumb length, function, and sensation, they require multiple operations, limit the joint space, have a poor aesthetic outcome, and donor site morbidity such as fractures and long-term recovery [5].

So, new techniques (one-stage osteoplastic reconstruction) have been discovered to evade the need for a second-stage procedure [3,5,6]. Immediate osteoplastic reconstruction can be done with a reverse-flow radial forearm osteocutaneous flap or autologous bone grafting using the amputated parts of the thumb. According to a previous study, forearm osteocutaneous flaps are a valid alternative when a patient has a normal pre-op Allen’s test. The radial artery perforators and antebrachial cutaneous nerve are preserved during dissection and then ligated to be transferred with the flap to reconstruct the thumb with a good sensation which obviates the need for a second-stage neurosensory island flap later on. In a proportion of cases, on the other hand, radial bone fractures, wound healing complications and skin graft loss at the donor site are reported [5].

Similar to our patient, two previous studies had undergone immediate osteoplastic reconstruction with the amputated parts of the thumb as an autologous bone graft followed by soft tissue coverage using a fasciocutaneous radial forearm flap, dorsal metacarpal flap, or groin flap [3,5,6]. In our opinion, osteoplastic reconstruction by taking the advantage of the amputated parts of the thumb is a perfect alternative with many advantages. What is interesting about this technique is its capability of preserving the thumb length and function which has been the most paramount digit without causing donor site complications. Hereby, we add to the literature another successful case of thumb reconstruction using skeletonized amputated phalanges after a failed thumb replantation.

Despite our patient having a functional thumb and pursuing his work with an acceptable range of motion, he refused to undergo the last stage which is the neurovascular flap operation and accepted his insensate thumb. As a result, we think that patients who underwent this procedure will have an acceptable range of motion which let them pursue their work and refuse to undergo a second-stage neurovascular flap. Previous studies suggested that patients with an insensate thumb, who do not undergo neurosensory reconstruction, are more vulnerable to develop bone resorption than others because they will exert extravagant pressure on the distal bone graft [6]. Counterintuitively and fortunately, the hand X-ray of our patient 11 months post-operatively did not show bone resorption in the thumb bone graft.

## Conclusions

Reconstruction of thumb amputation by using an autologous skeletonized bone graft of the amputated phalanges after a failure of immediate replantation results in efficient thumb function with natural length and acceptable range of motion, shorter hospitalization time, and decreased donor site morbidity. However, further studies should be done regarding the long-term results of this technique.
